# Transient splenial lesion as late complication of COVID-19 infection

**DOI:** 10.1055/s-0042-1755213

**Published:** 2022-11-09

**Authors:** Ali Koksal, Yasemin Ogul, Veysel Ayyildiz, Hayri Ogul

**Affiliations:** 1 Ankara Private Bayindir Hospital, Ankara, Turkey.; 2 Duzce Public Health Center, Duzce, Turkey.; 3 Suleyman Demirel University, Medical Faculty, Department of Radiology, Isparta, Turkey.; 4 Duzce University, Duzce, Medical Faculty, Department of Radiology, Turkey.

**Figure 1:**
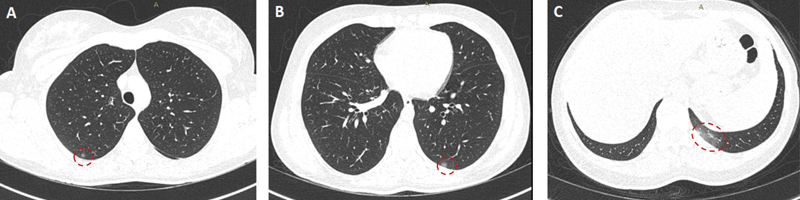
( **A–C** ) Axial thorax CT during the presentation scans show ground glass opacities (circles) in both lung parenchyma.

**Figure 2:**
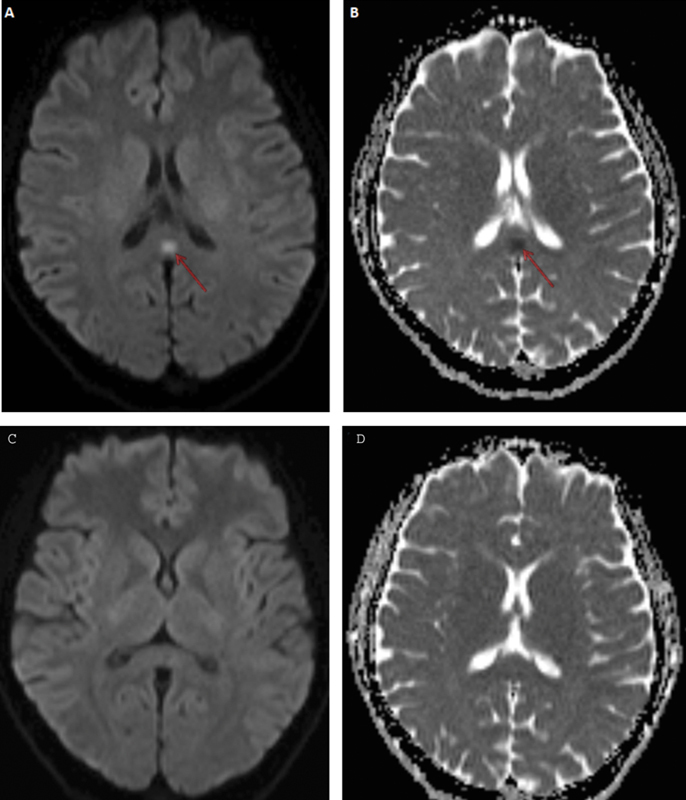
( **A** and **B** ) One month after successful therapy, diffusion weight imaging (DWI) shows significant diffusion restriction with reduced apparent diffusion coefficient (ADC) in the lesion. ( **C** and **D** ) One month after first MRI scan, DWI and ADC map reveal completely resolution of the lesion.

A 33-year-old woman presented with breathing discomfort, cough, and fever. The real-time reverse-transcriptase polymerase-chain-reaction (rRT-PCR) analysis was positive for coronavirus disease 2019 (COVID-19). The computed tomography (CT) scan showed ground glass opacities in lung parenchyma ( Figure 1A-C ). The patient was treated with favipiravir. One month after discharge, the magnetic resonance imaging (MRI) scan showed a lesion in the corpus callosum ( Figure 2A–B ). The imaging results were compatible with a transient splenial lesion. The patient was completely recovered after 1 month, without any specific treatment. Control MRI showed complete resolution of the lesion ( Figure 2C-D ). We thought that the splenial lesion was caused by the coronavirus infection; COVID-19 infection presenting with transient splenial lesion in an adult patient has been reported in only a few cases. 1 2 3
